# Characterization of antigen-specific CD8+ memory T cell subsets in peripheral blood of patients with multiple sclerosis

**DOI:** 10.3389/fimmu.2023.1110672

**Published:** 2023-05-04

**Authors:** Pen-Ju Liu, Ting-Ting Yang, Ze-Xin Fan, Guo-Bin Yuan, Lin Ma, Ze-Yi Wang, Jian-Feng Lu, Bo-Yi Yuan, Wen-Long Zou, Xing-Hu Zhang, Guang-Zhi Liu

**Affiliations:** ^1^ Department of Neurology, Beijing Anzhen Hospital, Capital Medical University, Beijing, China; ^2^ Department of Neurology, Beijing Tiantan Hospital, Capital Medical University, Beijing, China

**Keywords:** multiple sclerosis, peripheral blood mononuclear cells, memory T cells, oligodendrocyte glycoprotein, pentamer

## Abstract

**Background:**

Increasing evidence indicates the importance of CD8^+^ T cells in autoimmune attack against CNS myelin and axon in multiple sclerosis (MS). Previous research has also discovered that myelin-reactive T cells have memory phenotype functions in MS patients. However, limited evidence is available regarding the role of CD8^+^ memory T cell subsets in MS. This study aimed to explore potential antigen-specific memory T cell-related biomarkers and their association with disease activity.

**Methods:**

The myelin oligodendrocyte glycoprotein (MOG)-specific CD8^+^ memory T cell subsets and their related cytokines (perforin, granzyme B, interferon (IFN)-γ) and negative co-stimulatory molecules (programmed cell death protein 1 (PD-1), T- cell Ig and mucin domain 3 (Tim-3)) were analyzed by flow cytometry and real-time PCR in peripheral blood of patients with relapsing-remitting MS.

**Results:**

We found that MS patients had elevated frequency of MOG-specific CD8^+^ T cells, MOG-specific central memory T cells (T_CM_), MOG-specific CD8^+^ effector memory T cells (T_EM_), and MOG-specific CD8^+^ terminally differentiated cells (T_EMRA_); elevated granzyme B expression on MOG-specific CD8^+^ T_CM_; and, on MOG-specific CD8^+^ T_EM_, elevated granzyme B and reduced PD-1 expression. The Expanded Disability Status Scale score (EDSS) in MS patients was correlated with the frequency of MOG-specific CD8^+^ T_CM_, granzyme B expression in CD8^+^ T_CM_, and granzyme B and perforin expression on CD8^+^ T_EM_, but with reduced PD-1 expression on CD8^+^ T_EM_.

**Conclusion:**

The dysregulation of antigen-specific CD8^+^ memory T cell subsets, along with the abnormal expression of their related cytokines and negative co-stimulatory molecules, may reflect an excessive or persistent inflammatory response induced during early stages of the illness. Our findings strongly suggest positive regulatory roles for memory T cell populations in MS pathogenesis, probably *via* molecular mimicry to trigger or promote abnormal peripheral immune responses. Furthermore, downregulated PD-1 expression may stimulate a positive feedback effect, promoting MS-related inflammatory responses *via* the interaction of PD-1 ligands. Therefore, these parameters are potential serological biomarkers for predicting disease development in MS.

## Introduction

Multiple sclerosis (MS) is a chronic inflammatory demyelinating disease of the central nervous system (CNS). The disease frequently has a relapsing-remitting course and, in the later phases, tends to cause irreparable and severe neurological disability. Although its etiology remains uncertain, it is widely speculated that autoreactive T cell responses directed against CNS myelin are responsible for its pathogenesis (1). Importantly, several studies have found that MS patients have different memory phenotypes of myelin-reactive T cells.

Conventional CD4^+^ and CD8^+^ memory T cells can be divided into CCR7^+^CD45RA^−^ central memory T cells (T_CM_), CCR7^−^CD45RA^−^ effector memory T cells (T_EM_), and CD45RA^+^CCR7^−^ terminally differentiated cells (T_EMRA_), with district homing and effector properties (2–4). T_CM_ and naïve cells home to secondary lymphoid tissues in physiological circumstances, whereas T_EM_ and T_EMRA_ traffick to non-lymphoid organs that are inflamed and exert effects such as stimulating interferon (IFN)-γ secretion and causing potent cytotoxicities. T_CM_, in contrast, are longer-lived with a greater proliferative capacity (5, 6).

Mounting evidence indicates that CD8^+^ T cells have a significant role in autoimmune CNS attack in MS. CD8^+^ T cells are accumulated in white matter lesions. These cells frequently outnumber CD4^+^ T cells at this location, and are in a quiet neibourhood close to oligodendrocytes and demyelinated CNS axons (7–9), the latter of which are believed to produce early neurological symptoms (10). CD8^+^ T cells are present in immune cell infiltrates in the early phases of MS cortical demyelinating lesions (11). Strikingly, CD8^+^ T cells with an effector-memory phenotype have been found to accumulate in the MS lesions, exhibiting inflammatory and cytotoxic potential due to enhanced expression of granzyme B and interferon (IFN)-γ (12, 13). In parallel with these findings, we previously reported that MS patients display elevated CD8^+^CCR7^+^CD45RA^−^ T_CM_, which tends to decrease after treatment with the immunomodulatory agent IFN-β1α (14). Together, these findings strongly suggest that a skewed distribution of autoreactive CD8^+^ memory T-cell subsets is involved in the disease pathogenesis, given that an increased frequency of circulating CD4^+^ T_EM_ has been demonstrated in MS patients after specific antigen-driven stimulation. Notably, human leucocyte antigen (HLA)-A*03:01 is associated with about a two-fold increase in risk of developing MS—independent of HLA-DR2 (15–17).

However, further research is still required to understand the distribution of memory T-cell subsets in the illness and the potential contribution of abnormal T-cell homeostasis to the pathophysiology of inflammation. Thus, we analyzed circulating antigen-specific memory T-cell subsets using the pentamer, HLA-A*03:01-RVVHLYRNGK (myelin oligodendrocyte glycoprotein (MOG)_46-55_ peptide) and its related cytokines, to determine if these cell populations are correlated with disease activity.

## Patients and methods

### Subjects

Twenty-four patients with definite MS (five men and 19 women; mean age 35.2 ± 5.3 years) were included. All patients experienced relapsing-remitting MS (RRMS) and had never been using immunosuppressive medications, including glucocorticosteroids, for more than 6 months prior to the study. Five of these RRMS patients were chosen for serial examination while receiving treatment with teriflunomide (Aubagio^®^, Sanofi, Paris, France). Patients underwent clinical neurological examination including expanded disability status scale (EDSS), blood sampling before and after 2 and 4 weeks of treatment (18).

Twenty-three patients with atherothrombotic stroke (6 men and 17 women; mean age 36.7 ± 5.9 years) were recruited as “other neurological diseases” (OND) controls. Twenty-four healthy individuals participated as the healthy controls (“HC”, six men and 18 women; mean age 35.0 ± 6.5 years). All participating subjects were Han Chinese and tested positive for the A3 allele *via* HLA-A genotyping using polymerase chain reaction (PCR) sequence-specific primers (PCR-SSP). At the Department of Clinical Chemistry, whole blood cell counts and leucocyte differential analysis, were measured for patients and HC.

### Sample collection

Blood samples that had been heparinized were gathered between 9 and 12 AM. Blood samples from teriflunomide-treated individuals were taken before, and after 2 and 4 weeks of therapy as part of a serial trial.

### FACS-isolation of antigen-specific CD8^+^ memory T-cell subsets

To examine the expression of multiple cytokines (perforin, granzyme B, IFN-γ) and checkpoint receptor members ((programmed cell death protein 1 (PD-1) and T-cell Ig and mucin domain 3 (Tim-3)) in the antigen-specific CD8^+^ memory T-cell subsets, PBMCs were isolated by density gradient centrifugation and resuspended in phosphate buffer saline (PBS) containing 2% fetal calf serum. Thereafter, CD8^+^ T_CM_, T_EM_, and T_EMRA_ were isolated with allophycocyanin (APC)-labelled Pro5MHC Pentamer [HLA-A*03:01 MOG_46-55_ pentamers-RVVHLYRNGK] (Proimmune, Oxford, UK), phycoerythrin (PE)-cy7-labelled anti-CCR7 (eBioscience, San Diego, CA), APC-cy7-labelled anti-CD8 (BD Pharmingen, San Diego, CA, USA), and peridinin chlorophyll protein (PerCP)-Cy5.5-labelled-anti-CD45RA (Tonbo Biosciences, San Diego, CA) monoclonal antibody (MoAb) *via* a flow sorter (Becton Dickinson, San Jose, CA, USA).

### Flow cytometry

Antigen-specific CD8^+^ memory T-cell subsets were characterized in peripheral blood mononuclear cells (PBMCs) of the patients and controls by assessing PD-1 and Tim-3 expression *via* four-color direct fluorescence staining and flow cytometry using a FACScan (BD Biosciences, San Jose, CA, USA). After washing with PBS (0.5% BSA, pH 7.2), the cells were resuspended in PBS, to a final concentration of 1 × 10^6^ cells/mL. The following MoAb were added to 1 × 10^6^ cells following the manufacturer’s instructions: APC-cy7-labelled CD8; APC-labelled Pro5 MHC Pentamer [HLA-A*03:01 MOG_46-55_ pentamers-RVVHLYRNGK]; PE-cy7-labelled anti-CCR7; PerCP-Cyanine5.5-labelled-anti-CD45RA, Super Bright (SB)645-labelled anti-PD-1 (Thermo Fisher Scientific, Waltham, MA, USA); SB600-labelled anti-Tim-3 (Thermo Fisher Scientific). After 30-min incubation at room temperature, the cells were washed with PBS, fixed with 1% paraformaldehyde, and finally analyzed using a FACScan.

To perform intracellular cytokine staining, 1 × 10^6^ cells were incubated with the Cell Stimulation Cocktail (Tonbo Bioscience) for 4–5 h at 37°C in complete RPMI. Intracellular staining with eFluor 450-labelled anti-perforin (Thermo Fisher Scientific), Brilliant Violet (BV)510-labelled anti-granzyme B (BD Biosciences), and BV711-labelled anti-IFN-γ (BD Biosciences) MoAbs was completed following fixation/permeabilization according the manufacturer’s instructions (Cytofix/Cytoperm, BD Biosciences).

### RNA preparation and cDNA synthesis

RNA isolation was performed using RNeasy^®^ Mini Kit (Qiagen, Hilden, Germany), according to standard protocol. Reverse transcription was conducted with HiScript III 1st Strand cDNA Synthesis Kit (Nanjing Vazyme Biotech Co. Nanjing, China) using random hexamers and primer containing 50 µM oligio (dt). The process was carried out at 25°C for 10 min, 48°C for 30 min, and 95°C for 5 min on a T100 PCR system (Bio-Rad Laboratories, Hercules, CA, USA).

### Real-time PCR

Perforin, granzyme B, IFN-γ, PD-1, and Tim-3 mRNA were quantified using cDNA-specific primers (Sangon Biotec, Shanghai, China) as described elsewhere (**Table 1**) (19, 20). Twenty-five nanograms of cDNA and 200 nM forward and reverse primers were added to the PCR reactions using the AceQ Universal SYBR qPCR Master Mix (Nanjing Vazyme Biotech, Nanjing, China). β-Actin was chosen as the endogenous control. Real-time PCR was conducted using an ABI PRISM 7500 sequencing detector (Applied Biosystems, Foster City, CA, USA). Perforin, granzyme B, IFN- γ, PD-1, Tim-3, and 18 S PCR conditions were: hold at 50°C for 90 s, then 95°C for 10 min, followed by 40 cycles at 95°C for 15 s, 60°C for 1 min, and 72°C for 45 s.

**Table 1:** Primers used in the study.

Gene	Sense primer (5’-3’)	Antisense primer (5’-3’)
Perforin	GCAATGTGCATGTGTCTGTG	TTACCCAGGCTGAGTACTGCT
Interferon-γ	GCATCGTTTTGGGTTCTCTTGGCTGTTACTGC	CTCCTTTTTCGCTTCCCTGTTTTAGCTGCTGG
Granzyme B	GGGGAAGCTCCATAAATGTCACCT	TACACACAAGAAGGCCTCCAGAGT
PD-1	CAGGATGGTTCTTAGACTC	TACCAGTTTAGCACGAAG
Tim-3	CAGATACTGGCTAAATGGG	CTTGGCTGGTTTGATGAC
β-actin	ATCTGGCACCACACCTTCTACATTGAGCTGCG	CGTCATACTCCTGCTTGCTGATCCACATCTGC

### Statistics

Age, disease course, EDSS, and blood leukocyte count data are shown as means ± standard deviation. Memory T-cell subsets, surface and intracellular expression of cytokines, PD-1, and Tim-3 mRNA data are shown as medians with range. Categorical variables (sex) are expressed as percentages. Normal distribution data were analyzed using one-way ANOVA or a Pearson correlation test. Non-normal distribution data were analyzed using Kruskal-Wallis analysis or a Spearman correlation test. Categorical variables were analyzed utilizing a chi-square test. Receiver operating characteristic (ROC) curve analysis was conducted for quantitative MOG-specific CD8^+^ T_CM_, MOG-specific CD8^+^granzyme-B^+^ T_CM_ or T_EM_, MOG-specific CD8^+^perforin^+^ T_EM_, and MOG-specific CD8^+^ PD-1^+^ T_EM_ frequency, followed by the calculation of area under the ROC curve (AUC). *P* < 0.05 was deemed statistically significant.

## Results

### Clinical and laboratory data

**Table 2** shows the clinical and laboratory data gathered from patients during blood sampling. There were no remarkable differences among the three groups.

**Table 2:** Baseline characteristics of patients with multiple sclerosis (MS), other neurological disease (OND), and healthy controls (HC).

	MS (n=24)	OND (n=23)	HC (n=24)	*P* value
Age (years)	35.2 ± 5.3	36.7 ± 5.9	35.0 ± 6.5	0.533
Sex (male / female)	15/19	16/17	16/18	0.904
Disease course (years)	2.5 ± 1.3	–	–	–
EDSS	1.9 ± 1.4	–	–	–
HLA-A*03:01 [n (%)]	24 (100%)	23 (100%)	24 (100%)	0.999
Leukocyte (× 10^3^/μl)	5.70 ± 1.75	6.72 ± 1.62	6.53 ± 1.56	0.086
Neutrophils (× 10^3^/μl)	3.76 ± 1.28	4.39 ± 1.08	4.36 ± 1.44	0.162
Lymphocytes (× 10^3^/μl)	1.63 ± 0.54	1.62 ± 0.61	1.84 ± 0.48	0.298
Monocytes (× 10^3^/μl)	0.35 ± 0.08	0.41 ± 0.11	0.39 ± 0.13	0.101
Eosinophils (× 10^3^/μl)	0.07 ± 0.51	0.18 ± 0.14	0.11 ± 0.08	0.201
Basophils (× 10^3^/μl)	0.02 ± 0.01	0.03 ± 0.01	0.02 ± 0.01	0.092

Data are mean ± SD. EDSS, The Expanded Disability Status Scale.

### Memory T-cell subsets

**Table 3** shows the comparison of MOG-specific CD8+ T cells, naïve T cells and memory T cell subsets in peripheral blood between patients with MS,OND and HC.

**Table 3:** Comparison of MOG-specific CD8^+^ T cells and memory T cell subsets in peripheral blood between patients with multiple sclerosis (MS), other neurological disease (OND) and healthy controls (HC).

	Total CD8^+^ (%)	Naïve (%)	T_CM_ (%)	T_EM_ (%)	T_EMRA_ (%)
MS (n=24)	0.21 (0.12,0.37) **^∆∆^	16.7(5.46,35.30)*^∆^	11.95 (9.38,13.88) **^∆∆^	37.0 (25.33,44.98) **^∆∆^	0.25 (0,4.22) **^∆∆^
OND (n=23)	0.09 (0.03,0.11)	32.40(25.0,46.60)	3.14 (1.63,4.81)	12.30 (7.60, 28.60)	0 (0,0)
HC (n=24)	0.04 (0,0.1)	30.15(21.18,48.35)	4.65 (3.40,7.65)	10.90 (4.48,21.38)	0 (0,0)
*P* value	<0.0001	0.0184	<0.0001	<0.0001	0.0018

Data are median with range. **p < 0.01 or *p < 0.05 for post hoc comparison with HC; ^∆∆^p < 0.01 or ^∆^p < 0.05 for post hoc comparison with OND group. T_CM_, central memory T cells; T_EM_, effector memory T cells; T_EMRA_, terminally differentiated cells.

**Figure 1:**
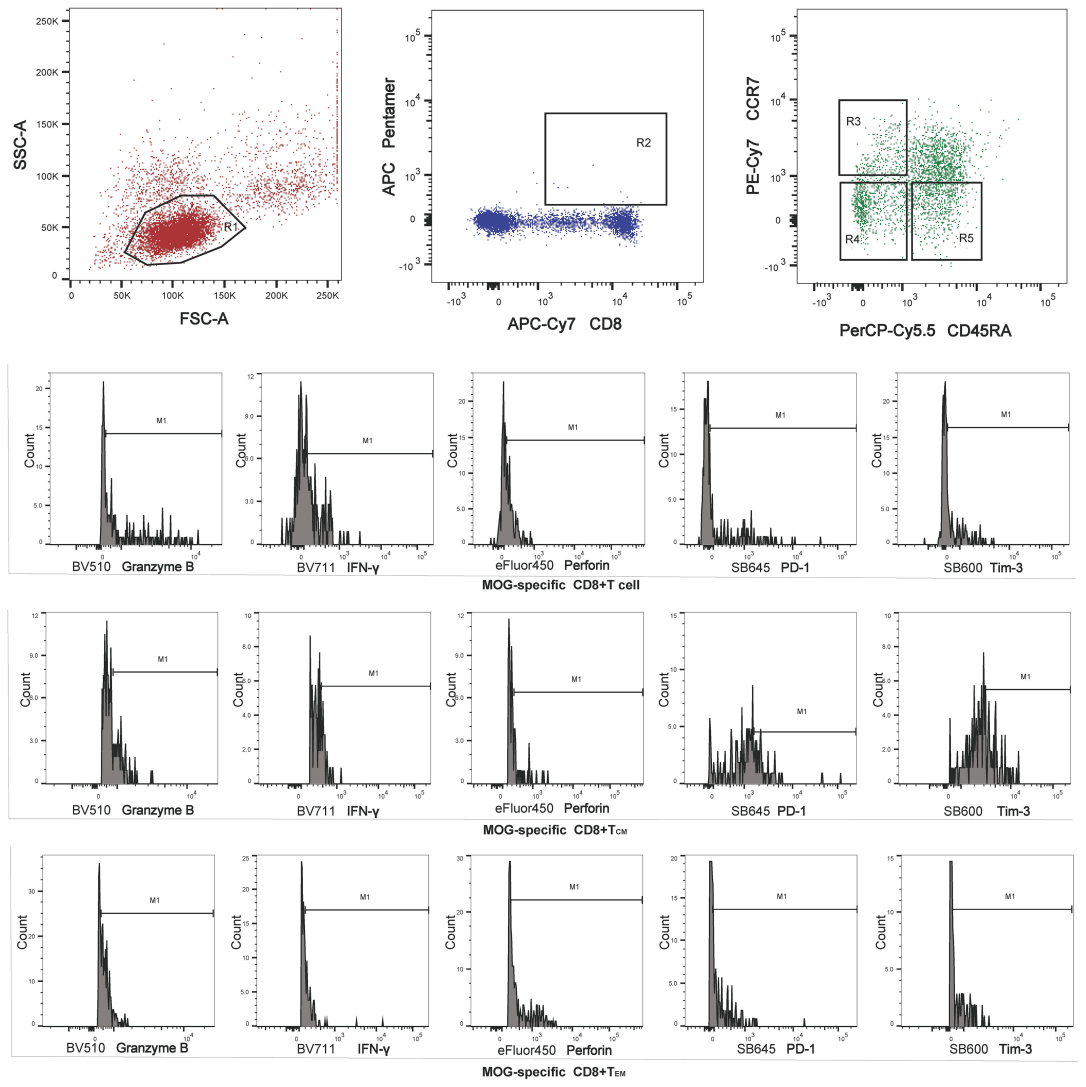
Region 1 (R1) was selected to set the mononuclear cell gate according to the forward light scatter (FSC) and side light scatter (SSC) properties. Region 2 (R2) was used to set the second gate, to separate MOG-specific CD8^+^ T cells for analysis of memory T cell subsets. Regions 3, 4, and 5 (R3–R5) were selected to set the central memory T cell (T_CM_), effector memory T cell (T_EM_) and terminally differentiated cell (T_EMRA_) gates, respectively, for perforin, granzyme B, interferon (IFN)-γ, programmed cell death protein 1 (PD-1), and T- cell Ig and mucin domain 3 (Tim-3) analysis.

**Figure 2:**
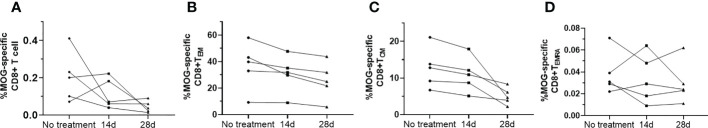
Serial study of the MOG-specific memory T-cell subsets **(A–D)** in the peripheral blood from five patients with multiple sclerosis (MS) before and after 14 d and 28 d of treatment with teriflunomide.

MS Patients had an increased proportion of MOG-specific CD8^+^ T cells, MOG-specific CD8^+^ T_CM_ (HLA-A*03:01/MOG_46-55_ pentamers^+^CCR7^+^CD45RA^−^), MOG-specific CD8^+^ T_EM_ (HLA-A*03:01/MOG_46-55_ pentamers^+^CCR7^−^CD45RA^−^), and MOG-specific CD8^+^ T_EMRA_ (HLA-A*03:01/MOG_46-55_ pentamers^+^CCR7^−^CD45RA^+^) compared to OND patients and HC *via* FACScan (**Figure 1**). In contrast, MS patients had a lower proportion of MOG-specific naive CD8^+^ T cells either OND patients or HC. After 2 and 4 weeks of therapy, five MS patients showed a tendency for a gradual decrease in MOG-specific CD8^+^ T_CM_ and T_EM_ (**Figures 2B, C**), while the remaining cell subsets displayed minor or irregular alternations (**Figures 2A, D**).

### Intracellular expression of perforin, granzyme B and IFN-γ, and surface expression of PD-1 and Tim-3

**Figure 3:**
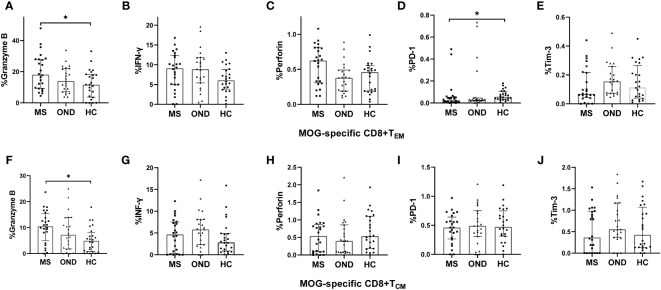
Peripheral blood expression of perforin, granzyme B, interferon (IFN)-γ, programmed cell death protein 1 (PD-1), and T- cell Ig and mucin domain 3 (Tim-3) on MOG-specific CD8+ effector memory T cells (TEM) **(A–E)** and CD8+ central memory T cells (TCM) **(F–J)**, in patients with multiple sclerosis (MS), those with other neurological disease (OND), and healthy controls (HC). Horizontal lines: medians. * P < 0.05, ** P < 0.01.

**Figure 4:**
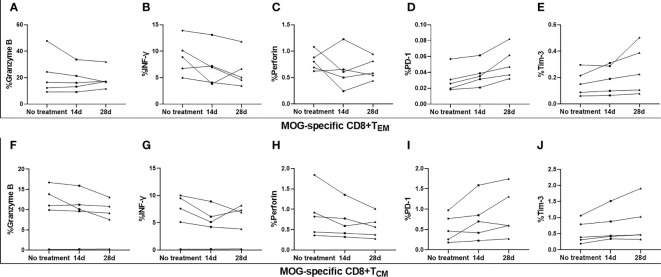
Serial analysis of perforin, granzyme B, interferon (IFN)-γ, programmed cell death protein 1 (PD-1), and T- cell Ig and mucin domain 3 (Tim-3) on MOG-specific CD8+ central memory T cells (TCM) and CD8+ effector memory T cells (TEM) in the peripheral blood of five patients with multiple sclerosis (MS) before and after 14 d and 28 d of treatment with teriflunomide **(A–J)**.

MS patients demonstrated elevated expression of granzyme B, and reduced expression of PD-1, on MOG-specific CD8^+^ T_EM_, compared to HC (**Figures 3A, D**), as well as increased expression of granzyme B on MOG-specific CD8^+^ T_CM_, when compared with HC (**Figure 3F**). Although there was a slight increase in perforin expression, no marked differences were found between these three groups (**Figures 3C, H**). OND patients demonstrated slightly higher expression of Tim-3 on MOG-specific CD8^+^ T_EM_ and CD8^+^ T_CM_ than HC, but did not reach statistical significance (**Figures 3E, J**). However, we did not measure the above cytokines or PD-1 and Tim-3 on CD8^+^ T_EMRA_, owing to the extremely low proportion of this cell population in peripheral blood. Five MS patients treated with teriflunomide exhibited a continuous increase in PD-1 expression on MOG-specific CD8^+^ T_EM_ after 2- and 4-weeks therapy (**Figure 4D**), while the expression of the remaining cytokines or co-stimulatory molecules presented slight or irregular changes (**Figures 4A-C, E-J**).

### Quantification of perforin, granzyme B, IFN-γ, PD-1, and Tim-3 mRNA expression

**Figure 5:**
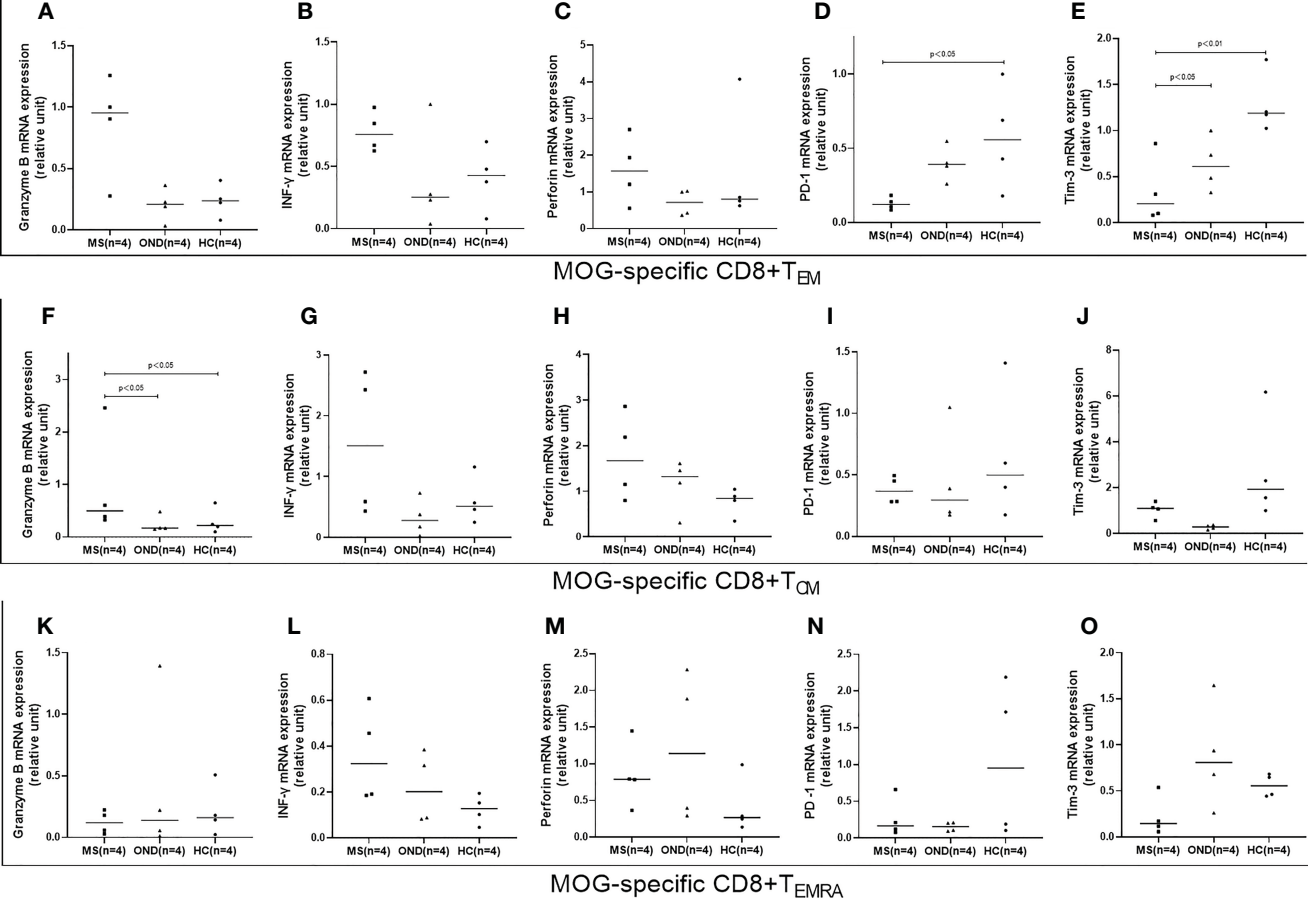
Peripheral blood mRNA expression of granzyme B, interferon (IFN)-γ, perforin, PD1, and TIM3 on CD8+ effector memory T cells (TEM) **(A–E)**, CD8+ central memory T cells (TCM) **(F–J)**, and terminally differentiated cells (TEMRA) **(K–O)**, in patients with multiple sclerosis (MS), those with other neurological disease (OND), and healthy controls (HC). Horizontal lines: medians.

Of the isolated CD8^+^ memory T cell subsets, MOG-specific CD8^+^ T_EM_ exhibited lower PD-1 and Tim-3 mRNA expression in MS patients than in HC, while PD-1 expression did not differ significantly between MS and OND patients (**Figures 5D, E**). MS patients displayed significantly higher mRNA expression of granzyme B in MOG-specific CD8^+^ T_CM_ than OND patients and HC (**Figure 5F**). However, no significant difference in above proinflammatory cytokines or PD-1 and Tim-3 mRNA expression in T_EMRA_ were found between MS patients and control groups.

### ROC of selected MOG-specific CD8^+^ T-cell subsets as well as their expression of cytokines and PD-1

**Figure 6:**
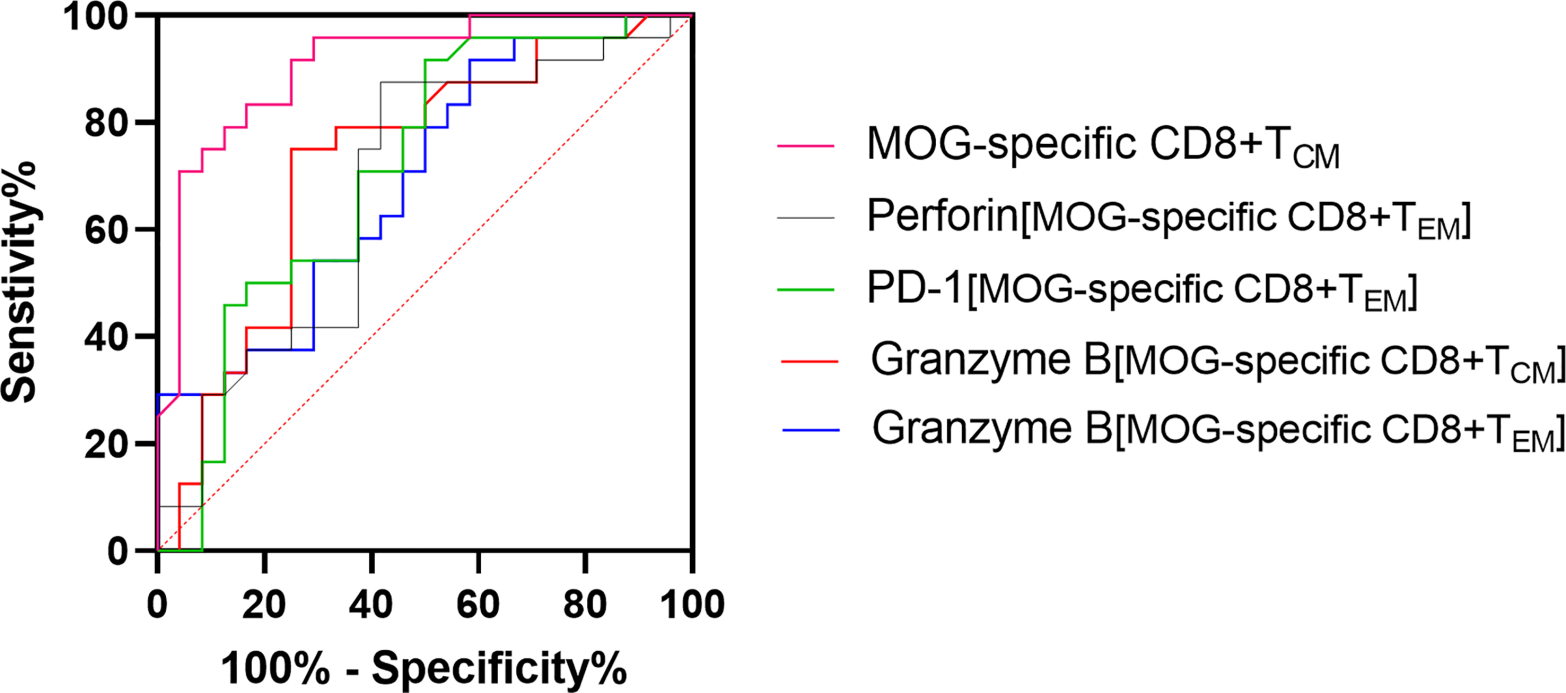
Receiver operating characteristic (ROC) curve analysis, in patients with multiple sclerosis (MS), of the frequency of MOG-specific CD8^+^ central memory T cells (T_CM_), MOG-specific CD8^+^granzyme-B^+^ T_CM_ or effector memory T cells (T_EM_), MOG-specific CD8^+^perforin^+^ T_EM_, and MOG-specific CD8^+^ PD-1^+^ T_EM_.

ROC curves were generated to calculate the AUC on the basis of the optimal cut-off value, as well as maximum sensitivity and specificity. For the MOG-specific CD8^+^ T_CM_ proportion, AUC was 0.9089 (optimal threshold cutoff, 6.65%; sensitivity, 91.67%; and specificity, 75%); for MOG-specific CD8^+^granzyme-B^+^ T_CM_, AUC was 0.6528 (optimal threshold cutoff, 7.605%; sensitivity, 66.67%; specificity, 75%); for MOG-specific CD8^+^granzyme-B^+^ T_EM_, AUC was 0.691 (optimal threshold cut-off, 4.364%; sensitivity, 100%; specificity, 29.17%); for MOG-specific CD8^+^perforin^+^ T_EM_, AUC was 0.678% (optimal threshold cutoff, 0.5885%; sensitivity, 58.33%; specificity, 87.5%); for MOG-specific CD8^+^ PD-1^+^ T_EM_, AUC was 0.6918 (optimal threshold cutoff, 0.0415%, sensitivity 75%, specificity, 70.83%) (**Figure 6**).

### Correlation analysis

In MS patients, EDSS score before treatment was correlated with the frequency of MOG-specific CD8^+^ T_CM_ (*r* = 0.421, *P* = 0.041); granzyme B expression in CD8^+^ T_CM_ (*r* = 0.507, *P* = 0.012); and, for CD8^+^ T_EM_, the expression of granzyme B (*r* = 0.512, *P* = 0.01), perforin (*r* = 0.446, *P* = 0.029), and PD-1 (*r* = −0.520, *P* = 0.009). EDSS score after treatment of teriflunomide was correlated with the frequency of MOG-specific CD8^+^ T_EM_ (*r* = 0.975, *P* = 0.033). Moreover, EDSS score after 4 weeks of treatment with teriflunomide was correlated with the granzyme B expression in MOG-specific CD8^+^ T_EM_ (*r* = 0.975, *P* = 0.033).

## Discussion

To the best of our knowledge, this is the first human-based research utilizing MHC pentamers to identify myelin antigen-specific CD8^+^ T cells and their memory T cell subsets in MS patients. As a result, MS patients had higher frequency of MOG-specific CD8^+^ T cells, MOG-specific CD8^+^ T_CM_, MOG-specific CD8^+^ T_EM,_ and MOG-specific CD8^+^ T_EMRA_, in contrast to a lower frequency of MOG-specific naïve CD8+ T cells; elevated granzyme B expression on MOG-specific CD8^+^ T_CM_; and, on MOG-specific CD8^+^ T_EM_, elevated granzyme B and reduced PD-1 expression. In MS patients, EDSS was correlated with the frequency of MOG-specific CD8^+^ T_CM_, granzyme B expression in CD8^+^ T_CM_, and granzyme-B and perforin expression on CD8^+^ T_EM_, but with reduced PD-1 expression on CD8^+^ T_EM_.

Our results indicate that MS patients have a skewed distribution of antigen-specific CD8^+^ memory T cell subsets, with more T_CM,_ T_EM_, and T_EMRA_, and fewer naive CD8^+^ T cells, than HC. These findings validate the derangement of these cell populations during the inflammatory process in MS. Together with our previous report revealing elevated IL-15 release into circulation (14), our current findings suggest that the differentiation of naïve cells is what causes the rise in CD8^+^ TCM and TEM because IL-15 is generally considered a central regulator of primary and memory antigen-specific CD8^+^ T cell production (21, 22). Interestingly, patients with atherosclerotic stroke and HC do not differ in the frequency of antigen-specific CD8^+^ T cells and their memory T cell subsets, since the former category of patients exhibits CD4^+^ memory T cells and CD8^+^ T cells in the atherosclerotic tissue from their carotid arteries (23). Hence, further research is required to elucidate this.

Several sphingosine 1-phosphate (S1P1) receptor antagonists, such as fingolimod (FTY720), have been commonly used for the treatment of RRMS; these antagonists selectively retain CCR7^+^ naïve T cells and T_CM_, and particularly autoreactive Th17 cells, within the secondary lymphoid organs (24–26). Nonetheless, the exact role of CD8^+^ T cell subsets such as T_CM_ and T_EM_ in MS remains elusive. However, in our recently published pilot study, adoptive transfer of autoreactive CD8^+^ T_CM_ into Rag-1−/− mice failed to induce EAE symptoms or EAE-related pathology (27). Notably, a lower proportion of memory CD8^+^ T cell subsets (particularly effector memory and T_EMRA_) has been observed in patients with untreated RRMS than in HC, probably due to inherent (i.e., genetically determined) defects rather than a pathophysiological effect of MS (28, 29). In our experiments, despite an irregular change in total MOG-specific CD8^+^ T cells in MS patients, MOG-specific CD8^+^ T_CM_ and CD8^+^ T_EM_ exhibited a decreasing trend at 14 and 28 d post-treatment with teriflunomide, a well-known immunosuppressant agent (30). Together with a recent MS study revealing markedly lower IFN-γ and tumor necrosis factor-α levels on T_EMRA_ and T_EM_ following 12 months of teriflunomide use (31), our results indicate that these cell populations play a positive regulatory role in disease pathogenesis, presumably *via* a mechanism of molecular mimicry to trigger or promote abnormal peripheral immune responses and, consequently, aggravate neuroinflammation, and myelin and axonal damage in the brain. The positive correlations that we observed here between disease severity and the frequencies of MOG-specific CD8^+^ T_CM_ and MOG-specific CD8^+^ granzyme B^+^ T_CM_ or T_EM_ further support this view. More importantly, these markers are potentially valuable in defining RRMS and secondary progressive MS (SPMS), because growing evidence suggests the involvement of distinct memory T cell subsets in different forms of MS and/or at different disease stages (29, 32, 33).

As negative co-stimulatory molecules, Tim-3 and PD-1 are expressed on the cell surface and negatively modulate the immune response; their signaling impairs functional activities of CD8^+^ T cell, eventually leading to CD8^+^ T cell exhaustion, particularly in chronic viral infection and tumors (34, 35). Co-expression of Tim-3 and PD-1 are characteristic of the most severely exhausted CD8^+^ T cell subset (36, 37). Blockade of Tim-3 and PD-1 pathway can reverse this exhaustion and rescue the T-cell function (38, 39). However, it is difficult to determine their function on CD8 ^+^ T cells, since TIM-3 is implicated in both T cell exhaustion and activation (35, 40–44). Here, MS patients showed a significant reduction in PD-1 and slight reduction in Tim-3 surface expression on MOG-specific CD8^+^ T_EM_, compared to HC. This suggests that the molecule dysregulation in CD8^+^ memory T cells might be more prevalent in CD8^+^ T_EM_ than in CD8^+^ T_CM_. However, these results should be interpreted with caution, because the expression of PD-1 and Tim-3 are insufficient to define the function of CD8^+^ T cells in the absence of their ligands. Nevertheless, more efforts are needed to explore their expression as well as the effects of these two costimulatory pathways on CD8^+^ T cells in our future study.

The reduced PD-1 and Tim-3 mRNA expression in MOG-specific CD8^+^ T_EM_ that we observed in MS patients further substantiates our findings. Consistent with our results, a previous study (45) reported that CD4^+^ and CD8^+^ T cells stimulated by myelin basic protein demonstrated significantly higher PD-1-positive cell frequency in stable than in acute MS. Tim-3 activation, on the other hand, stimulates the formation of effector T cells as evidenced by the acquisition of an activated effector phenotype, elevated cytokine secretion, higher proliferative activities, and a transcription program linked with the differentiation of human antigen-specific CD8 T cells (42). Indeed, there was a trend towards increased expression of PD-1 on CD8^+^ T_EM_ during 4 weeks of treatment with teriflunomide. Taken together, these findings for MS suggest aberrant PD-1 and Tim-3 co-stimulation in CD8^+^ T_EM_ rather than in CD8^+^ T_CM_. PD-1 may reduce the inflammation caused by local cell–cell interactions, by halting co-stimulation of the host immune cells such as lymphocytes, or by suppressing apoptotic signaling *via* interaction with PD-1 ligands (40, 46). Tim-3 may exert complex regulatory effects on the immunoactivity of CD8^+^ memory T cell subsets in different stages of MS. Together with our ROC analysis of abnormal antigen-specific memory T cell subsets, our findings strongly suggest that dysregulated PD-1 and Tim-3 co-stimulation are implicated in MS pathogenesis, and may therefore be useful for assessing disease severity.

## Conclusion

In conclusion, in MS patients, we observed remarkable upregulation of antigen-specific CD8^+^ T_EM,_ T_EMRA_ and T_CM_, with elevated intracellular expression of granzyme B and reduced expression of PD-1 in MOG-specific CD8^+^ T_EM_. This may implicate a persistent chronic CD8^+^ memory T cell-mediated inflammatory response, potentially induced in the early stages of this disorder. More strikingly, MS severity was at least partially reflected in the elevated MOG-specific CD8^+^ T_CM_ frequency and granzyme B and perforin expression, and reduced PD-1 expression. These are therefore potential serological biomarkers for predicting the development of MS. Nevertheless, further studies, such as those with a prospective cohort design, are required. Given that other disorders (e.g., oncological malignancies) also demonstrate dysregulated CD8^+^ memory T cells, it is necessary to evaluate their specificity, sensitivity, cytotoxicity, and negative co-stimulation to demonstrate whether these markers might be specific for MS. Other issues should be addressed, such as their prognostic vs. non-prognostic value in predicting acute episodes in patients diagnosed as clinically isolated syndrome (CIS). Our findings strongly suggest positive regulatory roles for memory T cell populations in MS pathogenesis, probably *via* molecular mimicry to trigger or promote abnormal peripheral immune responses. Furthermore, downregulated PD-1 expression may stimulate a positive feedback effect, promoting MS-related inflammatory responses *via* the interaction of its ligands (PDL1), although the expression of PD-1 is not sufficient to define the function of CD8^+^ T cells in the absence of its ligand. Our discovery of antigen-specific CD8^+^ T cell subsets and PD-1 expression in MS paves the way for identifying potential MS biomarkers and, more importantly, for exploring a novel treatment approach against this disease through intervention in the PD-1–PDL1 pathway.

## Data availability statement

The raw data supporting the conclusions of this article will be made available by the authors, without undue reservation.

## Ethics statement

The studies involving human participants were reviewed and approved by Ethics Committee of Beijing Anzhen Hospital, Capital Medical University. The patients/participants provided their written informed consent to participate in this study.

## Author contributions

P-JL and G-ZL wrote the article. G-ZL designed the research. P-JL and T-TY performed the research. Z-XF and Z-YW analyzed the data. G-BY, LM, W-LZ, J-FL, X-HZ, and B-YY contributed new reagents/analytical tools. All authors contributed to the article and approved the submitted version.
